# Withdrawal statement: [Liu, X., Qiao, L., Zhang, H., Zhang, C., Ding, R., Kang, J., Liu, Y., Lin, J. and Yan, X. (2023), Argonaute 2 regulates LPS‐induced inflammation in microglial BV2 cells by targeting Cadm1. FEBS Open Bio. Accepted Author Manuscript].

**DOI:** 10.1002/2211-5463.13606

**Published:** 2023-07-12

## Abstract

The above article, published online on 4 April 2023 in Wiley Online Library (wileyonlinelibrary.com) as an Accepted Article (https://doi.org/10.1002/2211‐5463.13606) has been withdrawn by agreement between the authors, the journal Editor‐in‐Chief Miguel A. De la Rosa, and John Wiley and Sons. The withdrawal has been agreed due to a dispute over authorship and the conceptualization of some of the data.

